# Risk Factors for Delayed Leptomeningeal Dissemination in Choroid Plexus Papillomas: A Systematic Review and Illustrative Case

**DOI:** 10.3390/curroncol33020114

**Published:** 2026-02-13

**Authors:** Orlando De Jesus, Ricardo J. Fernández-de Thomas, Cesar Carballo-Cuello, Bryan Clampitt

**Affiliations:** 1 Section of Neurosurgery, Department of Surgery, University of Puerto Rico, Medical Sciences Campus, San Juan 00936, Puerto Rico; cesar.carballo@upr.edu; 2 Center for Neurosciences, Department of Neurosurgery, Mennonite Hospital, Caguas 00725, Puerto Rico; ricardo.fernandez3@upr.edu; 3 Morsani College of Medicine, University of South Florida Health, Tampa, FL 33602, USA; baclampitt@usf.edu

**Keywords:** carcinomatosis, choroid plexus papilloma, leptomeningeal, pathology, risk factors, seeding

## Abstract

Purpose: Choroid plexus papilloma (CPP) is a rare, benign intraventricular tumor that typically has an excellent long-term survival rate after gross total resection. Nevertheless, some patients develop delayed leptomeningeal dissemination (LMD) years after the initial diagnosis, a phenomenon that remains poorly understood. The authors encountered a case of CPP with delayed LMD 14 years after resection, which prompted this systematic review to identify potential risk factors. Methods: A systematic review was conducted according to PRISMA guidelines to identify CPP reports with subsequent delayed LMD. Studies of atypical CPP, choroid plexus carcinoma, or concurrent LMD at diagnosis were excluded. Extracted variables included demographics, tumor location, extent of resection, recurrence, proliferative index, and latency to dissemination. Results: Thirty patients developed delayed LMD after initial diagnosis. Seventeen patients underwent gross total resection, ten underwent subtotal resection, and three were unreported. Delayed LMD occurred with tumor recurrence in 14 patients. Histological transformation was observed in nine patients: eight progressed to aCPP and one to CPC. The extent of resection, recurrence, and tumor transformation were not significantly associated with delayed LMD. Conclusions: The pathogenesis and optimal treatment strategy of this phenomenon remain unclear. No significant risk factors for delayed LMD development in CPP were identified. Future studies incorporating molecular characterization are needed to clarify the mechanisms of LMD in patients with CPP and to improve risk stratification, underscoring the importance of lifelong surveillance and the integration of molecular profiling in clinical management.

## Simple Summary

Choroid plexus papilloma is a benign intraventricular tumor with an excellent long-term survival rate after gross total resection. However, some patients develop delayed leptomeningeal dissemination years after the initial diagnosis, a phenomenon that remains poorly understood. A systematic review was conducted to identify reports of patients with subsequent delayed leptomeningeal dissemination. The review identified thirty patients who developed delayed leptomeningeal dissemination after initial diagnosis. The extent of resection, recurrence, and tumor transformation were not significantly associated with delayed leptomeningeal dissemination. The pathogenesis and optimal treatment strategy for this phenomenon remain unclear, as we could not identify any significant risk factors for delayed leptomeningeal dissemination in choroid plexus papilloma.

## 1. Introduction

Choroid plexus tumors (CPTs) are rare neoplasms derived from the choroid plexus epithelium, accounting for less than 1% of all intracranial neoplasms and 2–4% of pediatric brain tumors [1,2]. In adults, these tumors are primarily located within the fourth ventricle, whereas in children, they are usually found within the lateral ventricles [1,3]. The World Health Organization (WHO) classification categorizes CPTs into three grades: choroid plexus papilloma (CPP, grade I), atypical CPP (aCPP, grade II), and choroid plexus carcinoma (CPC, grade III). CPP is a benign tumor that resembles normal choroid plexus. While occasional mitoses may be present, CPP does not demonstrate cytological atypia or necrosis. The treatment of choice is gross total resection (GTR) [1,3,4]. Histology is a significant predictor of overall survival (OS) for CPTs, with CPP carrying the most favorable prognosis [4,5,6].

DNA methylation profiling has refined the classification of CPTs based on their clinical behavior. In 2016, Thomas et al. identified three subgroups: cluster 1 (pediatric CPP and aCPP of mainly supratentorial location), cluster 2 (adult CPP and aCPP of mainly infratentorial location), and cluster 3 (pediatric CPP, aCPP, and CPC of supratentorial location) [7]. A few years later, Capper et al. subdivided CPTs into plexus tumors subclass adult, subclass pediatric A, and subclass pediatric B [8]. More recently, CPTs have also been stratified into three epigenetic risk groups: supratentorial pediatric low-risk, infratentorial adult low-risk, and supratentorial pediatric high-risk [9]. These molecular and epigenetic classifications provide important insights into the heterogeneity of prognosis and may help explain variability in recurrence and LMD risk.

Despite these advances, the clinical behavior of CPP remains poorly understood. Although benign by histology, CPP can disseminate along cerebrospinal fluid (CSF) pathways. Leptomeningeal dissemination (LMD) may occur synchronously with the initial tumor diagnosis or be delayed, sometimes many years after tumor removal. Dissemination is most often identified when it co-occurs with the initial CPP diagnosis [1,10,11,12]. Synchronous LMD is thought to result from tumor cell shedding into CSF [13]. Delayed LMD may arise at the time of local recurrence; however, it can also develop without a recurrence [14]. Reported adverse prognostic features in CPP include a higher Ki-67 or MIB-1 index, an increased number of mitoses, and a lower proportion of tumor cells positive for S100 protein [11,15]. However, other potential factors remain poorly studied.

Delayed LMD is an uncommon but clinically significant complication that can develop years after apparent GTR of the initial tumor [1]. Its pathogenesis is not well defined, and identifying risk factors that predispose to this outcome is critical. We encountered a case of a CPP that developed delayed LMD 14 years after initial diagnosis. This observation motivated the present systematic review of patients with CPP who subsequently developed delayed LMD. This study aims to identify clinical, histological, and molecular risk factors and provide a more comprehensive understanding of long-term surveillance and management of LMD.

## 2. Methods

### 2.1. Study Design and Population

We conducted a systematic review of the literature on LMD in CPPs. Eligible patients were diagnosed with CPP at initial presentation and subsequently developed LMD. Cases of atypical CPP (aCPP) or choroid plexus carcinoma (CPC) at diagnosis were excluded. The objective was to identify risk factors that may contribute to the development of delayed LMD in CPP. This review was registered in the Prospective Register of Systematic Reviews (PROSPERO; registration number 1110261).

### 2.2. Search Strategy and Data Collection

A systematic review was conducted in accordance with the Preferred Reporting Items for Systematic Reviews and Meta-Analyses (PRISMA) guidelines (Supplementary Figure S1) [16]. Two investigators independently searched MEDLINE/PubMed and Scopus for articles describing LMD in patients with CPP, screened titles and abstracts, and removed duplicates. Non-human cases were excluded, and only articles written in English, French, or Spanish were considered.

The search strategy used a combination of Medical Subject Headings (MeSH) and keywords, including “choroid plexus papilloma,” “metastases,” “disseminated,” “dissemination,” “diffuse,” “leptomeningeal,” and “seeding.” The search period extended from database inception through May 2025. Two authors (R.F.D. and C.C.C.) independently selected eligible articles, and disagreements were resolved by the senior author (O.D.), who also performed data extraction.

### 2.3. Risks of Bias Assessment

Observational studies were appraised according to the STROBE guidelines, and case reports or case series were evaluated according to the CARE guidelines. The risk of bias was assessed independently by two reviewers (R.F.D. and C.C.C.) using the Joanna Briggs Institute checklists for case reports, case series, and case–control studies [17]. Discrepancies were resolved by consensus.

### 2.4. Selection and Data Extraction

**Figure 1:**
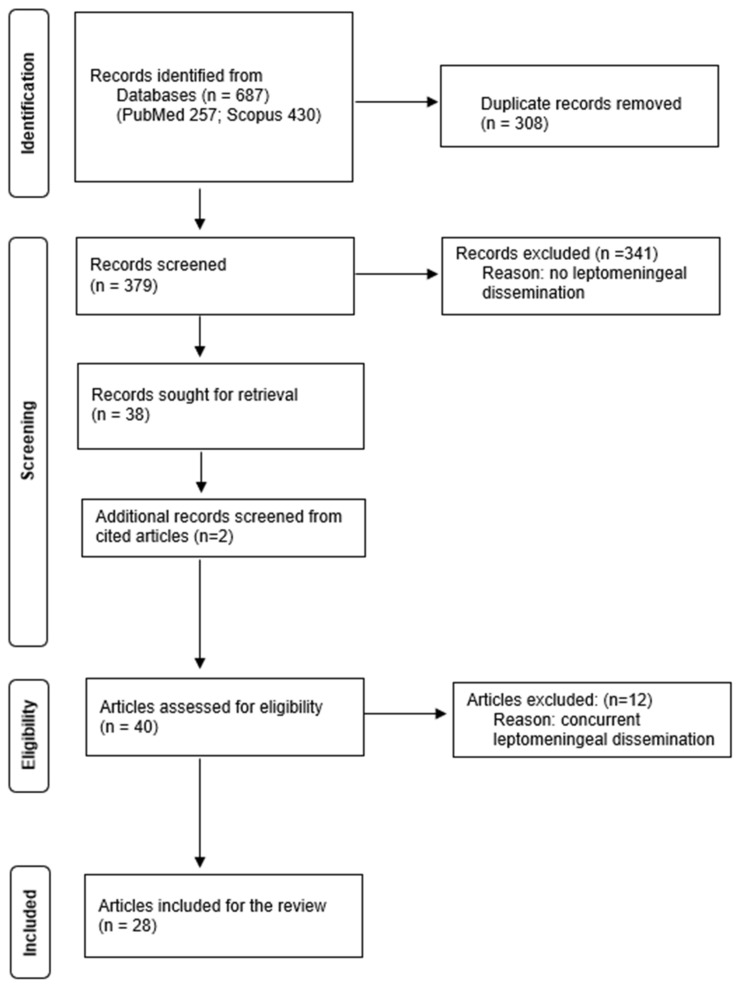
PRISMA flowchart for literature review screening and inclusion. Data added to the PRISMA template under the terms of the Creative Commons Attribution (CC BY 4.0) License (https://creativecommons.org/licenses/by/4.0/) (accessed on 16 July 2025).

A total of 379 articles published up to May 2025 were identified through database searches after removal of duplicates (Figure 1). These studies were screened to identify those that presented patients with CPP at initial presentation who subsequently developed LMD. Articles were excluded if patients had an initial diagnosis of aCPP or CPC. Studies describing single nodular recurrence or solitary metastasis were also excluded, as these did not represent LMD.

After screening, 38 articles were deemed eligible; 2 additional articles were identified through a reference review, yielding a total of 40 studies that reported on 47 patients who met the inclusion criteria. Articles were classified as concurrent (dissemination at the time of initial diagnosis) or non-concurrent (dissemination after initial treatment). Patients with LMD identified within 6 months of surgery were excluded from the non-concurrent group to reduce the risk of including perioperative seeding. Data were extracted with a standardized form and included: first author, year of publication, article title, study type, number of cases, patient age at initial diagnosis, sex, tumor location, extent of resection, mitotic activity, proliferation index, S100 expression, recurrence, tumor transformation, concurrency of LMD with the primary tumor, LMD location, and latency to LMD. Twelve articles reported concurrent cases, twenty-six reported non-concurrent cases. Two studies reported both types of LMD [1,12]. To identify risk factors for delayed LMD, the 28 articles describing 30 non-concurrent cases were included in the qualitative analysis. Risk factors considered were the extent of resection, recurrence, and tumor transformation. A new illustrative case report is also presented but was not included in the pooled analysis.

### 2.5. Statistical Analysis

Categorical variables were presented as frequencies and percentages, and normally distributed continuous variables were reported as the mean with standard deviation. Univariate analysis was performed to evaluate the extent of resection, recurrence, and tumor transformation as potential risk factors for delayed LMD. Time-to-event outcomes were estimated using the Kaplan–Meier method, with the median time and 95% confidence interval (CI) reported. Prespecified subgroup and predictor analyses were exploratory. Group comparisons used the chi-square test, hazard ratios (HR), and odds ratios as appropriate. Cox models were limited to clinically selected covariates, given the sample size, and model assumptions were checked. Two-sided *p*-values are reported without adjustment and should be interpreted as exploratory. Statistical analyses were conducted in Statistical Package for the Social Sciences, SPSS, version 29.0.0.0. A *p* < 0.05 was considered statistically significant.

## 3. Illustrative Case Report

**Figure 2:**
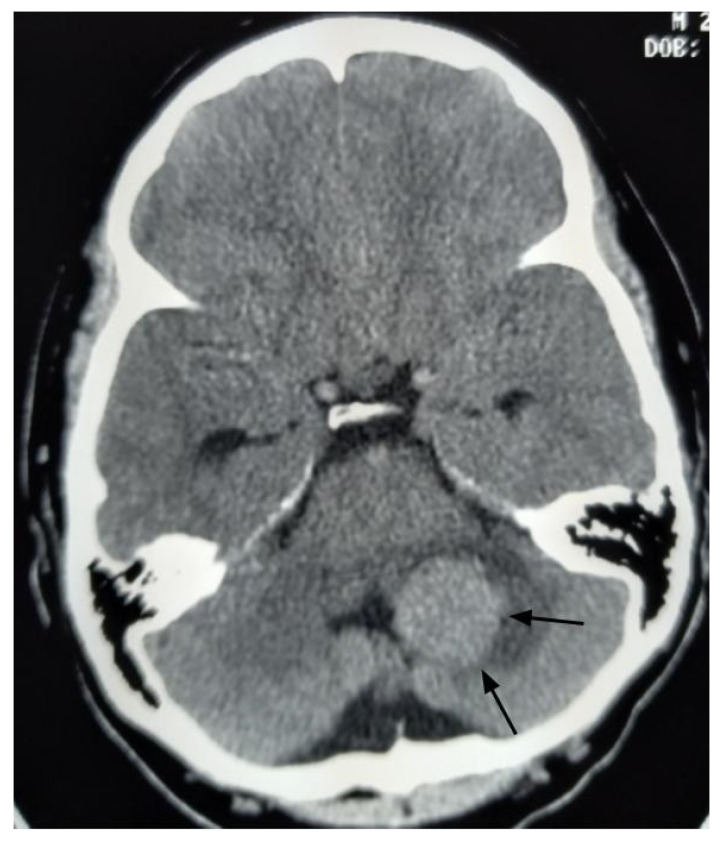
Axial head computed tomography with contrast image, performed before the initial surgery, reveals a large enhancing mass in the left cerebellum (black arrows).

A 26-year-old male presented to the emergency department with imbalance and severe headaches. Magnetic resonance imaging (MRI) was not available at the time of presentation. Due to obstructive hydrocephalus, emergent surgery was performed the same day using only a head computed tomography (Figure 2). The tumor was internally debulked to facilitate GTR. Pathology confirmed a fourth ventricular CPP exhibiting rare mitoses (up to 1 per 10 high-power fields) and a Ki-67 index of 5%. Postoperative brain MRI revealed no residual tumor or additional lesions.

Annual brain MRI surveillance was initiated. Two years later, a local recurrence occurred at the same location and was resected. Additional recurrences at the exact location were noted three years later and again two years thereafter. GTR was achieved in all three reoperations. Pathology consistently demonstrated CPP grade I with Ki-67 indices ranging from 2% to 5% and no mitotic figures. Annual brain MRI for the following six years revealed no further recurrence; however, spinal MRI was not obtained during this period, as it was not routinely performed in asymptomatic CPP adults.

**Figure 3:**
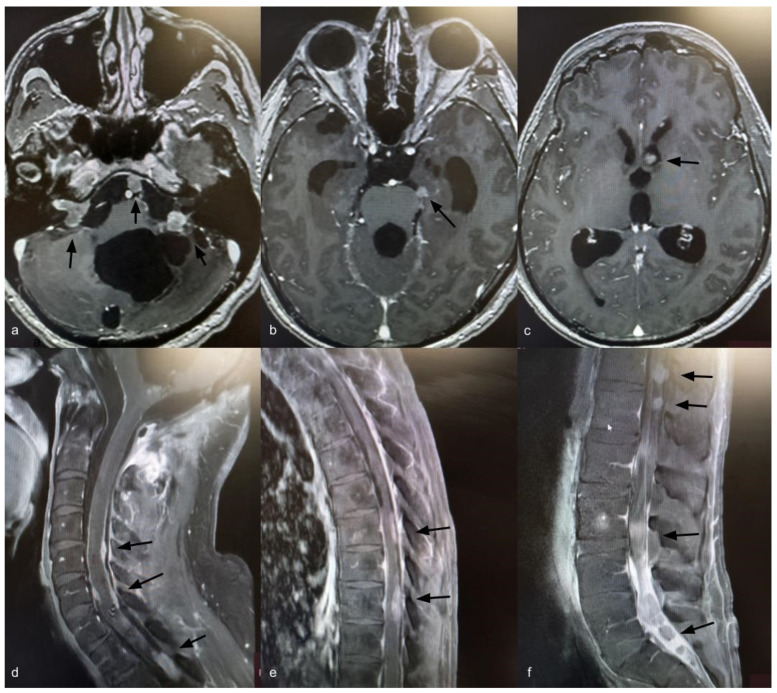
Axial T1-weighted brain MRI with gadolinium showing ventriculomegaly and multiple posterior fossa lesions at the right internal auditory canal, left cerebellopontine angle, prepontine cistern, and left frontal ventricular horn (**a**–**c**) (black arrows). Sagittal T1-weighted spine MRI with gadolinium showing leptomeningeal dissemination (**d**–**f**) (black arrows).

**Figure 4:**
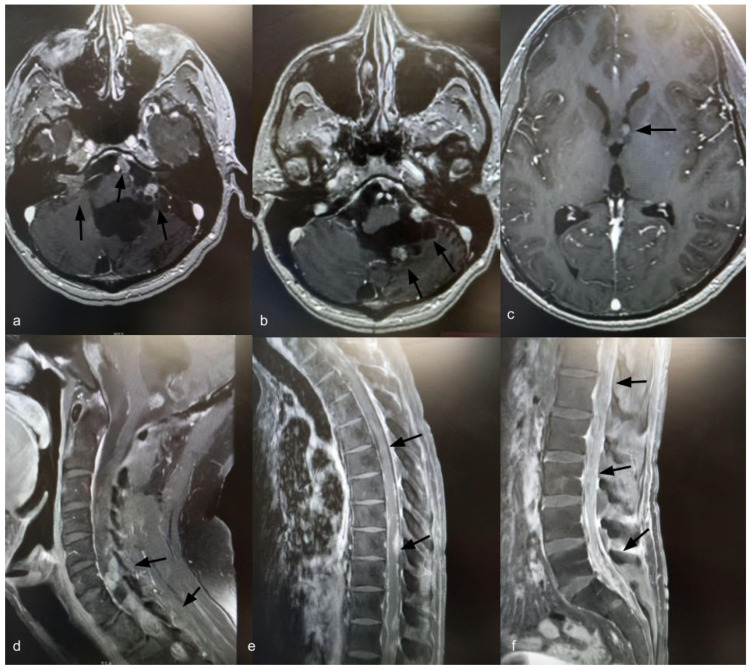
Axial T1-weighted brain MRI with gadolinium, 30 months after diagnosis of leptomeningeal dissemination, showing stable brain lesions (**a**–**c**) (indicated by black arrows). Sagittal T1-weighted spine MRI with gadolinium, 30 months after diagnosis of leptomeningeal dissemination, showing stable spinal enhancement and lesions (**d**–**f**) (indicated by black arrows).

Fourteen years after the initial surgery, at age 40, the patient developed acute right-sided hearing loss. Brain and spinal MRI demonstrated multiple posterior fossa lesions, diffuse spinal leptomeningeal enhancement, and multiple solid lesions throughout the entire spine (Figure 3), consistent with LMD. Surgery was not pursued, as no clinical benefit was anticipated. The patient underwent craniospinal irradiation comprising 30 sessions. At the three-month follow-up, MRI showed stable disease. He subsequently developed symptomatic communicating hydrocephalus requiring ventriculoperitoneal shunt placement. CSF cytology was negative for neoplastic cells. Six months after radiotherapy, with no radiological improvement, systemic chemotherapy was initiated with carboplatin (600 mg), etoposide (175 mg), and vincristine (2 mg), administered every 28 days for six cycles. Thirty months after the diagnosis of LMD, the patient remains alive with stable brain and spinal lesions with no evidence of new disease (Figure 4).

## 4. Results

The literature search identified 30 patients, reported across 28 articles, with delayed LMD arising from CPP [1,2,3,12,13,14,15,18,19,20,21,22,23,24,25,26,27,28,29,30,31,32,33,34,35,36,37,38]. The risk of bias assessment using the Joanna Briggs Institute criteria indicated that most studies had a low risk of bias; however, several reports demonstrated limitations due to the absence of histopathological information. Clinical and pathological features of the 30 patients are summarized in Table 1. The mean age at initial diagnosis was 34 ± 14.9 years. Most tumors (83%) originated in the fourth ventricle, while 13% arose in the lateral or third ventricle. The extent of resection was gross total in 17 patients, subtotal in 10, and unreported in 3. Mitotic activity was absent in 9 patients, reported as low in 1, and was not reported in 20. The Ki-67 index was reported in only a minority of cases, but when available, it was less than 4% in 6 patients. The mean latency to develop LMD was 8 ± 5.7 years. Delayed LMD occurred with local recurrence in 14 patients, while it ensued without recurrence in 16. Histological transformation occurred in nine patients: eight progressed to aCPP and one to CPC. In 17 patients, the tumor histology remained a CPP. In four patients, it was not reported, or they were not operated on. Two patients presented with multiple cystic lesions as the sole manifestation of their LMD.

Univariate analysis evaluated potential risk factors for delayed LMD, including the extent of resection (HR 0.641; 95% CI 0.287–1.433; *p* value 0.641), tumor recurrence (HR 1.012; 95% CI 0.477–2.147; *p* value 0.975), and tumor transformation (HR 1.432; 95% CI 0.628–3.261; *p* value 0.393). None of these variables was significantly associated with delayed LMD. Tumor location was not analyzed, as most tumors originated in the fourth ventricle. Histopathological details were incomplete in many reports and were therefore not assessed. Management of the LMD consisted primarily of surgical resection of one or more tumors in 73% of the patients. Adjuvant radiotherapy was administered to 33% of patients, and radiosurgery to 13%. Chemotherapy was administered to 6% of patients, including 2 cases of intrathecal therapy. One patient did not receive any treatment, as the LMD comprising leptomeningeal cysts did not progress over a seven-year follow-up. Overall survival could not be analyzed because one-third of the reports lacked clinical outcomes. Twenty-seven percent of the patients had died from the LMD. In 33%, the disease had not progressed, whereas in 7%, it had.

## 5. Discussion

### 5.1. Mechanisms of Dissemination

Leptomeningeal disease from primary solid tumors is a rare occurrence. Usually, it represents a late-stage complication, most often arising from malignant tumors, in which case it is referred to as leptomeningeal carcinomatosis [39]. Although CPP is a benign, slow-growing tumor, it can occasionally disseminate along CSF pathways, leading to drop metastases [13,29,31]. The mechanisms underlying LMD are unclear and poorly understood. The intraventricular location of CPTs may predispose them to seeding the neuraxis via the CSF, with dissemination more frequently observed in CPC [13]. The immediacy of the tumor to the ventricles contributes to its ability to disseminate through the CSF spaces. Iatrogenic spread during surgical manipulation has also been proposed as a potential mechanism [13]. Although not yet identified in CPP, molecular mechanisms underlying tumor cell proliferation, migration, motility, and adhesion in other primary brain tumors contribute to tumor cell dissemination [40,41,42]. Prognosis for patients with CPP and LMD varies widely, ranging from stable disease with mild symptoms to death within months of diagnosis [30].

### 5.2. Extent of Resection and Age

Older age and African American race have been associated with worse OS in CPPs [4]. Several studies reported the extent of resection as a significant predictor of OS [43,44]. However, in a larger series, Bhutada et al. recently found no difference in OS according to resection status [4]. Abdulkader et al. reviewed disseminated CPP in adults and found that 62% of patients underwent gross total resection, while 31% underwent subtotal resection [1]. Notably, LMD occurred more often among those reported to have a gross total resection [1]. In contrast, Nunes et al. observed that incomplete resection could increase the risk of LMD in CPTs overall, although this association was not apparent when stratified by histological subtype [12]. Our systematic review demonstrated that the extent of resection was not associated with delayed LMD. Abdulkader et al. indicated that the tumor that eventually became disseminated was diagnosed in most patients at age 30 or later, occurring slightly more frequently in females than in males [1]. Our review found that the mean age at initial diagnosis was 34 ± 14.9 years, with a mean latency to LMD development of 8 ± 5.7 years.

### 5.3. Histological Transformation

Malignant transformation of CPP with delayed LMD has been reported but remains rare. Transformation into aCPP has been described in several cases [2,14,19,26,29,32,34,36]. Transformation into CPC has occurred even less frequently [14,22]. Nonetheless, malignant progression of CPP can occur despite GTR of the initial tumor [14,22,45]. Abdulkader et al. found that in 65.5% of patients with disseminated CPP, the metastatic tumors retained the same WHO grade as the primary lesion [1]. In our study, histological transformation was observed in one-third of patients with delayed LMD; however, this finding was not statistically significant.

### 5.4. Cystic and Pseudo-LMD Phenomena

An unusual manifestation of CPP dissemination is the formation of leptomeningeal cysts. These lesions often demonstrate slow or absent growth, suggesting a benign natural history. Observation is generally recommended, with resection reserved for symptomatic or surgically accessible cysts [10,11,12]. In some cases, multiple cystic lesions can be the sole presentation of LMD [12,30]. By contrast, leptomeningeal enhancement around the brainstem or spine may resolve spontaneously following resection of CPP or aCPP and should not be misinterpreted as true dissemination [11,12,46,47]. This phenomenon has sometimes been referred to as “pseudo-LMD” [12]. It may be related to tumor-secreted vascular endothelial factors that induce a reactive meningeal enhancement [46]. Resolution of the meningeal enhancement after tumor removal supports this hypothesis [46]. Nunes et al. recommended delaying adjunct therapies until persistence of enhancement is confirmed on short-term follow-up, potentially avoiding overtreatment [12].

### 5.5. Therapeutic Approaches

Current treatments for LMD include radiation, chemotherapy, targeted agents, and immunotherapy [15,23,30,38,48]. However, standardized treatment algorithms are still lacking [13,30,31,38]. Zachary et al. reported an excellent response in a patient three years after craniospinal irradiation, with stable lesions [35]. However, Perez-Campos et al. observed no radiographic response to radiotherapy [38]. Conclusive evidence supporting a survival benefit from craniospinal irradiation in solid tumor leptomeningeal disease remains inconclusive [22,23,35,49,50]. The efficacy of adjuvant chemotherapy and radiation in patients with CPP dissemination remains inconsistent [13,15,23,26,30]. In our review, 33% of the patients who received chemotherapy were deceased, or the LMD had progressed; however, in 50%, it had not progressed. Intrathecal chemotherapy has shown no benefit [15,30]. The use of temozolomide has been scarce with unproven results [30]. However, targeted therapy and immunotherapeutic approaches hold potential for improving outcomes [39,50]. Although minimally invasive ablative techniques, such as laser interstitial thermal therapy, have not been used for LMD in CPPs, they may prove useful as an alternative local treatment strategy for selected patients with surgically challenging recurrences, progressive disease, or deeply located lesions [51].

### 5.6. Imaging and Surveillance

The appropriate follow-up interval for CPP remains unclear, as recurrences and metastases have been reported more than 15 years after gross total resection [1,29,33,37]. In pediatric patients with fourth ventricular tumors, whole-spine MRI is routinely performed at diagnosis to assess for drop metastases. Similar postoperative vigilance is warranted in both pediatric and adult CPP cases if a postoperative spinal imaging has not been obtained [34]. For patients with solely intracranial CPPs who underwent GTR, the routine usage of spinal imaging during follow-up vigilance is not recommended. However, for incompletely removed lesions, its use is advised. Nonetheless, for any patient presenting with a recurrent intracranial tumor, it should be mandatory. Several authors have suggested MRI surveillance of the entire neuraxis to detect local recurrences, spinal drop metastases, and LMD as early as possible, thereby enabling timely treatment [29,32,34].

### 5.7. Molecular Insights and Future Directions

Molecular studies may enable more accurate risk stratification of patients with CPP and provide insight into predicting which patients have tumors prone to disseminate or recur [52,53]. Feng et al. suggested that genetic events driving recurrence and dissemination may remain dormant for years, with recurrent tumors exhibiting the highest mutation burden [37]. Moreover, a TP53 mutation in a primary CPP may indicate a more aggressive biological behavior [37]. Future integration of genomic and epigenomic analyses may identify molecular signatures that distinguish CPPs at risk for delayed LMD from those with indolent behavior. Although it has not been used for choroid plexus leptomeningeal dissemination, CSF liquid biopsy to detect genomic alterations may be a potential tool for early detection, molecular characterization, and monitoring of dissemination [54,55,56]. A liquid biopsy is a potential option in certain cases where direct access to tumor tissue is difficult or risky due to its location. It can help to monitor treatment response, predict prognosis, and make therapeutic decisions [57,58,59,60].

### 5.8. Limitations

This study has several limitations. The analysis may be underpowered due to the limited number of cases available in the literature. Missing clinical outcomes in one-third of the reports restricted the OS analysis. Furthermore, the included cases span more than 50 years, during which treatment practices have evolved, potentially influencing outcomes. It should be recognized that molecular diagnostic techniques were incorporated into the routine diagnosis and classification of choroid plexus tumors only in the mid-2010s; consequently, many of the included cases predate the molecular era. In our illustrative case, surgery or a lumbar puncture for CSF cytology was not performed at the time of dissemination; thus, the actual tumor grade is uncertain. Nonetheless, this review contributes to understanding the potential risk factors for delayed LMD in CPP and highlights directions for future research.

## 6. Conclusions

Based on the 30 reported cases of CPP with delayed LMD, no significant clinical or pathological risk factors were identified. Delayed LMD was observed in both GTR and subtotal resection cases, with some association with tumor recurrence and transformation, suggesting multifactorial drivers. The pathogenesis of this phenomenon remains unclear, likely due to the heterogeneity of reported cases and the limited sample size. The optimal treatment strategy for CPP with LMD remains to be established, highlighting the importance of lifelong surveillance in clinical decision-making. Given the rarity of delayed LMD in CPP, international multicenter registries and molecular correlation studies will be crucial in improving risk stratification and guiding adjuvant therapy decisions.

## Figures and Tables

**Table 1:** Non-concurrent choroid plexus papilloma leptomeningeal dissemination.

References	Age */Sex	Location CPP	Resection	Time to LMD	Location LMD	M	Ki-67%	Transformation	Recurrence	Treatment for LMD	Outcome
Wilkins and Rutledge, 1961 [3]	56/M	4th V	GTR	5 y	B	NA	NA	NA	No	SR	D 2y
Wolfson and Brown, 1977 [18]	11/M	4th V	STR	5 y	S	NA	NA	No	No	SR	D 3y
Masuzawa et al., 1981 [19]	7/M	4th V	STR	4 y	S	low	NA	aCPP	No	SR	NA
Leys et al., 1986 [20]	40/M	4th V	GTR	9 y	B, Lat V	NA	NA	No	No	SR	D 1.5y
Girardot et al., 1990 [21]	30/F	CM	NA	5 y	4th V, LR	NA	NA	No	Yes	SR	NA
Niikawa et al., 1993 [22]	38/M	4th V	GTR	6 y	SS, CPA, S	NA	NA	CPC	Yes	SR, R	NP 8m
Shakespeare et al., 1997 [23]	27/F	4th V	STR	3 y	B, S	0	NA	No	Yes	R, C	NP 1.1y
Talacchi et al., 1999 [24]	38/M	4th V	GTR	5 y	SS, CPA	NA	NA	NA	Yes	RS	D 2y
Irsutti et al., 2000 [25]	48/F	4th V	GTR	8 y	SS	0	<4	No	Yes	SR	NP 6m
Valencak et al., 2000 [26]	33/F	4th V	STR	2 y	B, S	NA	NA	aCPP	Yes	SR, R, RS, C	NP 3y
Jagielski et al., 2001 [27]	50/M	4th, 3rd V	STR	4 y	B, S	0	NA	No	No	SR	D 1y
McEvoy et al., 2002 [28]	51/M	4th V	GTR	5 y	S, SS, B	0	low	No	No	SR	NA
Yu et al., 2006 [29]	30/M	4th V	GTR	19 y	S	NA	NA	aCPP	No	SR	NA
McCall et al., 2006 [30]	30/F	4th V	GTR	8 y	B, S	NA	NA	No	Yes	SR, C	NP 1.5y
McCall et al., 2006 [30]	22/F	4th V	GTR	5 y	S, SS	NA	NA	No	No	C	PD 4y
Ahn and Cho 2007, [13]	36/F	4th V	GTR	6 y	S, LR	0	NA	No	Yes	SR, C	NA
Ortega et al., 2007 [15]	20/F	4th V	GTR	6 y	S	0	1	No	No	C	D 2m
Kaptanoglu et al., 2007 [31]	51/F	4th V	GTR	7 y	S	NA	NA	No	No	SR	NP 1y
Menon et al., 2010 [2]	43/M	CPA	STR	4 y	B, S	NA	NA	aCPP	No	SR, R	NA
Palmer et al., 2010 [32]	55/M	4th V	NA	13 y	S, LR	NA	NA	aCPP	Yes	SR, R	NA
Al-Abdullah et al., 2011 [33]	35/M	4th V	GTR	16 y	B, CPA	NA	NA	No	No	NA	NA
Stuivenvolt et al., 2012 [34]	44/F	4th V	GTR	6 y	S	NA	NA	aCPP	Yes	SR, R	NP 1y
Dhillon et al., 2013 [14]	53/F	Lat V	GTR	4 y	3rd V, Lat V	0	1	aCPP, then CPC	Yes	SR, R	D 2y
Zachary et al., 2014 [35]	21/F	4th V	STR	8 y	B, S	0	NA	No	Yes	SR, R	NP 3y
Abdulkader et al., 2016 [1]	19/F	Lat V	STR	19 y	S, SS, 3rd V, 4th V	0	1	No	No	SR	P 1y
Abdulkader et al., 2016 [1]	27/M	4th V	GTR	25 y	B, LR	NA	NA	No	Yes	RS	D
Karthigeyan et al., 2021 [36]	32/F	4th V	GTR	6 y	S, LR	0	NA	aCPP	Yes	R	NP 1.2y
Feng et al., 2024 [37]	60/M	4th V	STR	17 y	S, LR	NA	3	No	Yes	SR, RS	NA
Perez-Campos et al., 2024 [38]	11/F	Lat V	NA	N/A	B	NA	NA	NA	No	SR, R	NA
Nunes et al., 2025 [12]	6/M	4th V	STR	4 y	B	NA	NA	NA	Yes	None	NP 7y
Current Study, 2026	26/M	4th V	GTR	14 y	B, S, Lat V, IAC	0	2–5	NA	Yes	R, C	NP 2.5y

* Age at initial diagnosis; B = brain, C = chemotherapy; CM = cisterna magna; CPP = choroid plexus papilloma; D = deceased; GTR = gross total resection; IAC = internal auditory canal; Lat = lateral; LMD = leptomeningeal dissemination; LR = local recurrence; m = months; M = mitoses; NA = not available; NP = no progression; PD = progressive disease; R = radiotherapy; RS = radiosurgery; S = spine; SR = surgical resection; SS = suprasellar; STR = subtotal resection; V = ventricle; y = years.

## Data Availability

The original contributions presented in this study are included in the article. Further inquiries can be directed to the corresponding author.

## Supplementary Materials

The following supporting information can be downloaded at https://www.mdpi.com/article/10.3390/curroncol33020114/s1, Figure S1: PRISMA Checklist.

## Abbreviations

The following abbreviations are used in this manuscript:

aCPP	atypical choroid plexus papilloma
CI	confidence interval
CPC	choroid plexus carcinoma
CPP	choroid plexus papilloma
CPT	choroid plexus tumor
CSF	cerebrospinal fluid
GTR	gross total resection
HR	hazard ratio
LMD	leptomeningeal dissemination
MRI	magnetic resonance imaging
PRISMA	Preferred Reporting Items for Systematic Reviews and Meta-Analysis
OS	overall survival
WHO	World Health Organization
